# Health study of 11,800 workers under occupational noise in Xinjiang

**DOI:** 10.1186/s12889-021-10496-3

**Published:** 2021-03-06

**Authors:** Shiyu Zhao, Dongkui He, Hanwei Zhang, Tingting Hou, Chengxin Yang, Wen Ding, Ping He

**Affiliations:** 1 Occupational Disease Prevention and Treatment Hospital of Xinjiang Uyghur Autonomous Region, Urumqi, 830091 Xinjiang China; 2 Department of Public Health, Medical College, Shihezi University, Shihezi, 832000 Xinjiang China; 3 The Third People’s Hospital of Xinjiang Uyghur Autonomous Region, Urumqi, 830091 Xinjiang China

**Keywords:** Occupational noise, Physical examination, Hearing, Blood pressure, Electrocardiogram

## Abstract

**Background:**

We investigated the health status of some workers exposed to occupational noise in Xinjiang, and explored the influencing factors of their health level. We aimed to determine the key protection groups of occupational noise hazards, which might provide the basis for the development of targeted noise prevention measures.

**Methods:**

We used descriptive analysis to investigate a total of 11,800 participants who underwent occupational health examination in Xinjiang Occupational Disease Prevention Hospital.

**Results:**

The hearing abnormality rate of noise exposure participants was 8.03%, which was higher in males than females (χ^2^ = 54.507, *p* < 0.05). The abnormal rate of high-frequency hearing threshold in Xinjiang minorities was lower than in Han nationality (χ^2^ = 11.780, *p* < 0.05), while the results of the electrocardiogram were reversed (χ^2^ = 9.128, *p* < 0.05). Differences in abnormal rates of blood pressure (χ^2^ = 149.734, *p* < 0.05), hearing (χ^2^ = 231.203, *p* < 0.05), and physical examination (χ^2^ = 360.609, *p* < 0.05) are statistically significant in different industries. The abnormal rates of blood pressure (χ^2^ = 67.416, *p* < 0.05) and hearing (χ^2^ = 49.535, *p* < 0.05) gradually decrease with the expansion of the enterprise scale. Logistic regression analysis showed that gender, nationality, age, enterprise size, and industry were closely related to pure tone audiometry examination abnormal rate.

**Conclusion:**

Workers of male, elder, in mine and small/medium enterprises should be the key populations to prevent occupational noise hazard. It is necessary to standardize occupational health management in enterprises, which helps to improve workers’ self-protection awareness and quality of life.

## Background

Production noise refers to the noise generated from machine rotation, gas emission, workpiece impact, and friction. With the increase in industry numbers and sizes, production noise has become a recognized public health problem in the world. More than 600 million workers worldwide have experienced levels of occupational noise above hazardous thresholds [1]. Approximately 16% of workers have lost hearing due to noise at work, and occupational noise deafness accounts for 16.7% of the total number of occupational patients in China [2]. Since the worker’s exposure to noise has decreased in some developed countries because of advanced technologies, the shift from agriculture to basic industry in developing countries is increasing together with the likelihood of occupational noise exposure [3]. Occupational noise exposure not only affects the auditory system but also impairs the functions of other organs. We need to reduce the productive noise and protect vulnerable groups together. The purpose of this study is to investigate the health status of some workers exposed to occupational noise in Xinjiang and try to understand the outcomes for individuals. Our results may provide evidence for the prevention and control of occupational noise hazards in the future.

## Methods

### Data sources and study participants

This study included 11,800 workers who underwent occupational health examinations at Xinjiang Occupational Disease Prevention and Control Hospital from January 1 to December 31, 2019, and whose occupational hazards were noise. All participants came from 168 companies, and the industries were divided into five categories (National Economy Industry Classification GB / T 4754—2017), three enterprises scale (Statistical Methods for the Classification of Large, Medium, Small and Micro Enterprises 201,175). All participants signed informed consent forms and the study was approved by the Medical Ethics Committee of The Third People’s Hospital of Xinjiang Uyghur Autonomous Region (No. XJSQ20200428–10).

### Clinical measurement

The occupational health examination program was conducted in accordance with the Chinese occupational health monitoring regulations (Occupational health monitoring technical specifications GB / Z 188–2014). The physical examination included blood pressure examination, electrocardiogram examination, pure tone hearing threshold test. There were two kinds of final results of occupational health examinations, abnormality or normal.

### Blood pressure examination (*BP*)

The examinee rested for at least 10 min in a quiet environment. The physical muscles of the subject were relaxed, the palms were extended upwards, the elbows were at the same level as the heart, the cuffs were flat against the skin, and the elasticity was moderate. The lower edge was 1 to 2 cm higher than the elbows. Three measurements were performed at an interval of 5 min, using an electronic sphygmomanometer (12 V8117; OMRON; China). The average of the measurement results was used. According to the World Health Organization standard classification, subjects with diastolic pressure > 90 and systolic pressure > 140 were defined as hypertension patients.

### Electrocardiogram examination (*ECG*)

Before the examination, workers rested in a supine position within 5-10 min. The electrocardiograph with conventional 12-lead electrocardiograms (ECG-1350; Nihon Kohden; Japan) was used to measure the electrocardiogram and was operated by a professionally trained doctor. The examinees were under supine measurement. The results were interpreted according to the international standard of ECG. All results were abnormal except for sinus heart rate.

### Pure tone audiometry examination (*PTA*)

The examinees were required to avoid noisy environments for up to 12 h before the test. All subjects who underwent pure-tone hearing tests used a pure tone audiometer (AD226; Interacoustics; Danish) in rooms with a background noise level of less than 25 dB. Both ears were tested using ascending pure tones at frequencies of 0.5, 1, 2, 3, 4, and 6 kHz. The test was repeated at least 3 times to determine the lowest signal strength as the final threshold for each ear. The average thresholds of 3, 4, 6 kHz were used to determine high-frequency hearing status. The binaural high-frequency average hearing threshold ≤ of 40 dB was treated as normal.

### Statistical analyses

Continuous data were shown as mean ± standard deviation. Student’s t-tests and ANOVA tests were analyzed by using independent-sample, LSD-t-tests were used to analyze Pairwise comparison between multiple groups. Qualitative data were analyzed by the Pearson χ2 test. Data were analyzed with IBM SPSS (ver 20). Differences would be considered significant if the *P*-value was < 0.05.

## Results

This study included 11,800 workers (from 168 companies) with a mean age of 35.0 (range 18 to 66) years. There were 10,626 males, accounting for 90.5%, and 1190 minority employees, accounting for 10.08% (Table 1**).**

**Table 1:** The socio-demographic characteristics of the workers

**Variable**	**n(%)**
**Gender**	
Male	10,626 (90.05%)
Female	1174 (9.95%)
**Nationality**	
The Han nationality	10,610 (89.92%)
Minorities	1190 (10.08)
**Industry**	
Mining	3005 (25.47%)
Manufacturing	6332 (53.67%)
EGW^a^	949 (8.04%)
Transportation^b^	856 (7.25%)
Others	658 (5.58%)
**Enterprise size**	
Large-scale	3005 (65.51%)
Mid-scale	633 (13.80%)
Small-scale	949 (20.69%)

^a^Electricity, gas and water production, and supply industries

^b^The companies participating in this study are all in the air transport industry

The abnormal rates of ECG, blood pressure, and PTA in participants was 36.42, 17.37% and, 8.02%, respectively. There were 80.23% of participants who had at least one abnormal physical examination result. The abnormal rates of high frequency hearing threshold in male participants were higher than females (*p* < 0.05), but the working-age were slightly longer than female participants (*p* < 0.05), but the difference was not obvious. The abnormal rates of the electrocardiogram in Han participants were lower than other ethnic minorities (*p* < 0.05), but the abnormal rates of high frequency hearing threshold were higher (*p* < 0.05)(Table 2**).**

**Table 2:** Analysis of occupational health examination results by gender and nationality

	Total	Gender				Nationality			
		Male	Female	t/χ^2^	*P*	Han	Minorities	t/χ^2^	*P-value*
**AGE** (Years, $\overline{x}$ ±*s*)	35.23 ± 9.75	35.25 ± 9.89	35.02 ± 8.47	0.760	0.451	35.27 ± 9.86	34.90 ± 8.84	1.214	0.232
**Working-age** (Years, $\overline{x}$ ±*s*)	6.65 ± 7.61	6.78 ± 7.78	5.47 ± 5.78	5.603	0.003	6.65 ± 7.56	6.74 ± 8.14	0.394	0.701
**ECG**									
Normal (n)	7502	6731	771	2.475	0.116	6793	709	9.128	0.003
Abnormal (n)	4298	3895	403			3817	481		
**PB**									
Normal (n)	9750	8765	985	1.474	0.225	8759	991	0.390	0.532
Abnormal (n)	2050	1861	189			1851	199		
**PTA**									
Normal (n)	10,853	9708	1145	54.507	<0.001	9728	1125	11.780	0.001
Abnormal (n)	947	918	29			882	65		
**Medical examination results**									
Normal (n)	2333	2106	227	0.156	0.693	7089	758	4.666	0.031
Abnormal (n)	9467	8520	947			3521	432		

Both age and working-age had effects on electrocardiogram, blood pressure, high-frequency hearing threshold, and abnormal rate of physical examination (*p* < 0.05), and age had a greater positive impact on it. Except for the abnormal rates of electrocardiogram (*p* < 0.05), the results of occupational health examination of participants of different industries and different enterprise sizes had statistical differences. There were significant differences between the working-age groups in all industries (*p* < 0.05); the comparison between the age distribution groups in all industries (except for the electricity, gas and water production and supply groups and other industry groups) had significant differences (*p* < 0.05) (Table 3).

**Table 3:** Analysis of occupational health examination results of workers in different industries

	Mining	Manufacturing	EGW	Transportation	Others	F/χ^2^	*P-value*
**AGE** (Years, $\overline{x}$ ±*s*)	41.97 ± 8.98	31.31 ± 8.07	39.18 ± 9.88^a^	33.16 ± 8.17	39.17 ± 9.36^a^	899.372	<0.001
**Working-age** (Years, $\overline{x}$ ±*s*)	11.85 ± 9.98	4.13 ± 4.45	8.33 ± 8.37	6.45 ± 6.84	5.09 ± 6.47	665.241	<0.001
**ECG**							
Normal (n)	1887	4052	627	523	413	6.269	0.180
Abnormal (n)	1118	2280	322	333	245		
**PB**							
Normal (n)	2314	5454	736	730	516	149.734	<0.001
Abnormal (n)	691	878	213	126	142		
**PTA**							
Normal (n)	2574	5996	876	805	602	231.203	<0.001
Abnormal (n)	431	336	73	51	56		
**Medical examination results**							
Normal (n)	504	1054	226	356	193	360.609	<0.001
Abnormal (n)	2501	5278	723	500	465		

^a^There was no statistical difference between the groups

Comparing the size of enterprises, there were significant differences in working-age (*p* < 0.05); the age distribution of participants in large enterprises and small and medium enterprises had significant differences (*p* < 0.05)(Table 4).

**Table 4:** Analysis of the results of occupational health examination of workers in different scale enterprises

	large-scale	Mid-scale	Small-scale	F/χ^2^	*P-value*
**AGE** (Years, $\overline{x}$ ±*s*)	33.53 ± 8.98	38.43 ± 10.17^a^	38.23 ± 11.02^a^	352.31	<0.001
**Working-age** (Years, $\overline{x}$ ±*s*)	6.66 ± 7.58	7.17 ± 7.83	4.54 ± 6.68	39.767	<0.001
**ECG**					
Normal (n)	4865	2125	512	2.509	0.285
Abnormal (n)	2806	1173	319		
**PB**					
Normal (n)	6490	2631	629	67.416	<0.001
Abnormal (n)	1181	667	202		
**PTA**					
Normal (n)	7152	2967	734	49.535	<0.001
Abnormal (n)	519	331	97		
**Medical examination results**					
Normal (n)	1482	650	201	11.213	0.004
Abnormal (n)	6189	2648	630		

^a^There was no statistical difference between the groups

Logistic regression analysis showed gender, nationality, age, enterprise size, and industry were related to PTA abnormal rate. Some factors were not related to PTA abnormal rate, such as working-age, PB, ECG. Male was at a higher risk of high-frequency hearing threshold abnormalities than female (OR = 0.302, 95% CI = 0.207–0.441, *p* = 0.000). Compared with minorities, the Han nationality had a higher risk of high-frequency hearing threshold abnormalities (OR = 0.668, 95% CI = 0.512–0.871, *p* = 0.003). Elder workers had a higher risk of abnormal hearing threshold (OR = 1.065, 95% CI = 1.057–1.074, *p* = 0.000). Workers worked in small-scale companies have a higher risk of high-frequency hearing threshold abnormalities than those in large and medium-scale companies. Workers worked in the mining industry have a higher risk of high-frequency hearing threshold abnormalities than those in the other industries (Table 5).

**Table 5:** Logistic regression analysis for the factors that associate with PTA abnormal rate

Variables	β	S.E.	Wald	OR	95%CI	*P*-value
**Gender** (Male vs Female)	−1.197	0.193	38.461	0.302	0.207–0.441	0.000
**Nationality** (Hans vs Minorities)	−0.403	0.135	8.859	0.668	0.512–0.871	0.003
**Age**	0.063	0.004	256.435	1.065	1.057–1.074	0.000
**Enterprise size**						
Small-scale			10.771			0.005
Mid-scale	−0.378	0.128	8.712	0.685	0.533–0.881	0.003
Large-scale	−0.441	0.137	10.419	0.643	0.492–0.841	0.001
**Industry**						
Mining			39.377			0.000
Manufacturing	−0.439	0.092	23.006	0.644	0.538–0.771	0.000
EGW	−0.667	0.139	22.901	0.513	0.391–0.674	0.000
Transportation	−0.497	0.165	9.067	0.608	0.440–0.841	0.003
Others	−0.520	0.161	10.421	0.595	0.434–0.815	0.001

*OR* Odds ratio, *CI* Confidence interval

## Discussion

It is obvious that the gender ratio of participants in this study is seriously unequal, and most of them are male (90%). According to the industry classification data of 186 enterprises, the large proportion of males is caused by the preference of mining and manufacturing industries for men in job selection and job demand.

In general, the medical examination results of occupational noise-exposed workers were relatively poor, and 80.23% of these participants had at least one abnormal medical examination result. The abnormal rates of both ECG (36.42%) and blood pressure (17.37%) were high in participants. This suggests that noise exposure may have a greater effect on the cardiovascular system, we should pay more attention to the cardiovascular disease of workers, find the early risk of disease, and improve the health of workers.

Earlier studies had found that females were more sensitive to hearing at higher frequencies than males, but the result was contrary in low-frequency areas. With age increasing, the auditory function of male decays faster. The overall hearing functions of females are better than males, and occupational hearing losses in males are always higher than in females [4]. There are no differences of genders in participants’ ECG, blood pressure, and physical examination results, except for the abnormal rate of binaural hearing threshold (male: 8.64% female: 2.47% total: 8.03% logistic regression analysis: OR = 0.302, 95% CI = 0.207–0.441, *p* = 0.000). This is consistent with the results of many studies in recent years. The cumulative occupational noise exposure is slightly higher in males than in females, which may also affect the medical results in addition to the gender differences. Therefore, we should focus on the results of occupational health inspections on male workers and increase the levels of protection for male workers. In 2018, Lin Daojian studied the hearing impairment rate (48.1%) of 2473 noise workers in Zhuhai City [5], and Qian Xuequan studied the hearing impairment rate (28.3%) of 639 noise workers in an oil field in Xinjiang [6]. However, the damage rate in this study is lower than the above literature. Possible reasons include improvement of equipment, workers’ protection plans, and their protection awareness.

Ethnic minorities (Caucasian race predominates), except the Han, make up 60% of the total population of Xinjiang. This is a regional characteristic, and their appearances and living habits are significantly different from those of the Han nationality compared with other provinces in China. This study has found the abnormal rate of the electrocardiogram in ethnic minorities is higher than Han nationality, but the result of hearing impairment is reversed. Luo Rui has analyzed the electrocardiograms of the ethnic minorities (the Caucasian ethnic group) and the Hans in a physical examination in Xinjiang. They found that the abnormal rate of ECG in the ethnic minorities was significantly higher than Hans, which was consistent with our results [7]. This is closely related to the customs and diet habits of ethnic minorities. Xinjiang ethnic minorities have a high salty diet with mainly pasta and meatbut eat less fresh vegetables and fruits. They are lack trace elements and folic acid which are at high risk for heart disease. We can infer that the noise factor is not the main reason for abnormal ECG of workers in Xinjiang. The results in 2013 by Themann have shown that hearing sensitivity decreased with pigmentation [8]. This conclusion may explain that minorities’ normal high-frequency hearing threshold ratio is still higher than Hans,s. The logistic regression analysis has shown that the risk of abnormal hearing threshold in minorities is 0.668 times of Hans, even if the protection in former groups may be relatively weak due to language problems. To sum up, we think it is less possibility for the race in the health effects of noise exposure.

The influence of aging in individuals is irresistible, which reduces the basic metabolism, organ senescence, and dysfunction. In this study, the noise exposure workers in the mining industry are the eldest, followed by those in electricity, heat and gas production and supply industries, other industries, and transportation, and final manufacturing. This is consistent with the detection rates of abnormal blood pressure and high-frequency hearing threshold. These abnormalities are possibly caused by age. Studies have shown that the effects of age and working-age accelerate age-related hearing loss, while there is a moderate degree of correlation between the accumulation of occupational noise exposure and age and hypertension [9]. Logistic regression analysis in our study has shown that older workers have a higher risk of abnormal hearing thresholds than youth, which is consistent with the above conclusions.

The mining and the production and supply of electric heating gas industries have a high incidence of noise exposure and diseases induced by noise. Our logistic regression analysis has shown the risk of abnormal hearing threshold in mining is highest, but the occupational noise exposure workers in other industries have high blood pressure and high frequency hearing threshold abnormalities. Although there is less occupational noise exposure in some industries, workers in these industries still suffer hearing loss and health effects because of no awareness of occupational noise exposure and inadequate protection. Kerns had found that 31% of medical and diagnostic laboratory workers were exposed to noise and have substantial hearing impairments, which was higher than in the mining and construction industries [10]. Therefore, it is necessary to evaluate the noise exposure of each industry and occupation and strengthen management on “low-risk” industries.

However, this study has found that the abnormal rate of blood pressure and high-frequency hearing threshold of exposed workers gradually decreases with the expansion of the size of the enterprise. The Logistic regression analysis has shown that workers working in small-scale companies have a higher risk of high-frequency hearing threshold abnormalities than those in large and medium-scale companies. Compared with large enterprises, small enterprises provide less prevention and education to workers, so that workers lack safety awareness and safety interventions [11, 12]. Our results are consistent with the above studies. We aim to promote occupational health in small and medium-sized enterprises to eliminate or reduce safety hazards in the work environment.

This study shows that workers who are male, elderly, or working in mining industries, or working in small-scale enterprises are vulnerable to noise damage and are also a key group for occupational noise protection. Enterprises in the mining industry should early protect hearing loss and noise-sensitive workers. Furthermore, multilingual training should be carried out to reduce the workers of the ethnic minorities from noise exposure. These populations will then get a better understanding of occupational hazards and their protection. Small businesses should also establish occupational health surveillance systems to promote sustainable development. This study analyzes the health effects of noise exposure on workers, which provides basic data for occupational health management in the region.

This study may have some limitations. OAE tests can identify minor damage for hearing loss due to noise. It is also more objective than PTA, so future studies will use PTA to overcome this shortcoming. The cross-sectional design may limit causality, which is also the limitation of our article. In the future, we can use biological monitoring and noise dosimetry to assess occupational exposure more accurately.

## Conclusion

This study shows that male, elderly, mining industries and small-scale enterprises are vulnerable to noise damage and are also key groups for occupational noise protection. Enterprises in the mining industry should early detect those persons with hearing loss and noise sensitivity. Multilingual protective training should be carried out to ensure that workers of the ethnic minorities can conveniently communicate and get hazard-protection knowledge. Occupational health surveillance systems should be established in small businesses soon for their sustainable development. Our results of noise exposure on health effects in different industries and populations will provide the basis for future occupational prevention and management.

## Data Availability

The datasets used during the current study are available from the corresponding author on reasonable request.

## Abbreviations

BP: Blood pressure examination
ECG: Electrocardiogram examination
PTA: Pure tone audiometry examination
EGW: Electricity, gas and water production and supply industries

## References

[1] Daniel E (2007). Noise and hearing loss: a review. J Sch Health.

[2] Nelson DI, Nelson RY, Concha-Barrientos M, Fingerhut M (2005). The global burden of occupational noise-induced hearing loss. Am J Ind Med.

[3] Fuente A, Hickson L (2011). Noise-induced hearing loss in Asia. Int J Audio.

[4] Hoffman HJ, Dobie RA, Losonczy KG, Themann CL, Flamme GA (2017). Declining prevalence of hearing loss in US adults aged 20 to 69 years. JAMA Otolaryngol Head Neck surg.

[5] Lin DJ, Wang HF, Mai WH, Wang H (2015). Analysis of abnormal results of occupational physical examination of 2473 workers exposed to noise. Chin Fore Med Res.

[6] Qian XQ, Guo WJ, Li LJ (2015). Effects of noise on workers’ health in an oil field in Xinjiang. Occup Health.

[7] Luo R, Zhang L (2018). Comparative analysis of physical examination ECG of kazak nationality and han nationality in a county. Guide Chin Mede.

[8] Themann CL, Byrne DC, Davis RR, Morata TC, Murphy WJ, Stephenson MR (2015). Early prognosis of noise-induced hearing loss: prioritising prevention over prediction. Occup Environ Med.

[9] Masterson EA, Tak SW, Themann CL (2013). Prevalence of hearing loss in the United States by industry. Am J Ind Med.

[10] Kerns E, Masterson EA, Themann CL, Calvert GM (2018). Cardiovascular conditions, hearing difficulty, and occupational noise exposure within US industries and occupations. Am J Ind Med.

[11] Jahangiri M, Azmon H, Daneshvar A (2019). Occupational health problems and safety conditions among small and medium-sized enterprises: a cross-sectional study in shiraz. Iran Ann Glob Health.

[12] Boustras G, Hadjimanolis A (2015). Management of health and safety in micro companies in Cyprus: results on ergonomic issues. Work..

